# Link prediction in multiplex online social networks

**DOI:** 10.1098/rsos.160863

**Published:** 2017-02-08

**Authors:** Mahdi Jalili, Yasin Orouskhani, Milad Asgari, Nazanin Alipourfard, Matjaž Perc

**Affiliations:** 1 School of Engineering, RMIT University, Melbourne, Victoria, Australia; 2 Department of Computer Engineering, Sharif University of Technology, Tehran, Iran; 3 Department of Computer Science, University of California, Riverside, CA, USA; 4 Department of Computer Science, University of Southern California, Los Angeles, CA, USA; 5 Faculty of Natural Sciences and Mathematics, University of Maribor, Maribor, Slovenia; 6 Center for Applied Mathematics and Theoretical Physics, University of Maribor, Maribor, Slovenia

**Keywords:** social networks, complex networks, signed networks, link prediction, machine learning

## Abstract

Online social networks play a major role in modern societies, and they have shaped the way social relationships evolve. Link prediction in social networks has many potential applications such as recommending new items to users, friendship suggestion and discovering spurious connections. Many real social networks evolve the connections in multiple layers (e.g. multiple social networking platforms). In this article, we study the link prediction problem in multiplex networks. As an example, we consider a multiplex network of Twitter (as a microblogging service) and Foursquare (as a location-based social network). We consider social networks of the same users in these two platforms and develop a meta-path-based algorithm for predicting the links. The connectivity information of the two layers is used to predict the links in Foursquare network. Three classical classifiers (naive Bayes, support vector machines (SVM) and K-nearest neighbour) are used for the classification task. Although the networks are not highly correlated in the layers, our experiments show that including the cross-layer information significantly improves the prediction performance. The SVM classifier results in the best performance with an average accuracy of 89%.

## 1. Introduction

Mining data gathered from online social networks is one of the key research topics in computer science. Online social networks such as LinkedIn, Facebook and Twitter play important roles in shaping and reflecting the social relations in modern societies. A social network can be modelled as a network with users as the nodes and their social relationships as edges. These edges can be friendship, collaboration, following or mutual interests. *Network science*, as an interdisciplinary science field, is now a mature branch of science with many applications in science and engineering [1,2]. Tools available in network science have been frequently applied to analyse social networks and study their statistical and dynamical properties.

The link prediction problem is one of the topics in network science that is relevant to social networks analysis and mining [3,4]. Social networks often consist of missing and spurious links, and predicting such links will help better understand their mechanisms [5,6]. The link prediction problem, in general, tries to understand the association between two nodes and answer interesting questions such as [3]: How does the pattern of association change over time? What are the general factors driving the associations? To what extent can one model the evolution of a network using features intrinsic to the network itself? One can use methods such as perturbation theory to study the extent that the links are predictable in networks [7].

The link prediction problem has significant applications in areas such as bioinformatics (link discovery and network reconstruction are major challenges of network biology and medicine [8–10]), e-commerce [11,12] and social networks technology [4]. A successful application in e-commerce is recommender systems where a list of potential items are recommended to users based on the history of user–item interactions (ratings (or purchase) history of users on items) and their contextual information [13–17]. The recommendation problem can be modelled as predicting forthcoming links from users to items. There is a rich literature on predicting missing links in networked structures, for a review, see [3]. Indeed, the link prediction problem has been a long-standing challenge in modern information science, and many algorithms (e.g. those based on statistical models or Markov chains) have been proposed in computer science. Recently, with the progress in network science and development of statistical tools to study networked structures, many research works have shown that the structure of a network can be effectively used to infer the association between the nodes, and to build models for predicting missing links [18]. Various link prediction algorithms have been proposed, including local/global similarity-based algorithms (assigning a similarity score for non-adjacent nodes), maximum-likelihood methods and probabilistic models [3]. One can also use network reconstruction methods as an alternative to link prediction [19].

Traditionally, a networked system has been modelled as a single-layer network by treating all its nodes from the same type and all edges on an equivalent footing. However, such a modelling approach can lead to incorrect description of some phenomena in many systems. Some real systems have multilayer structures [20,21]. An example of such structures is social networks where the actual relationships between the members take place inside different groups (layers), or in some cases on different platforms such as Facebook and Twitter [22]. The same set of individuals may have a particular connection structure in Facebook and different links in Twitter. Multilayer networks are often divided into two broad categories: multiplex and heterogeneous networks [20,21]. Multiplex networks are a class of multilayer networks for which the same type of nodes are connected through different types of links. Brain networks are multiplex in that the same neurons can be coupled through chemical and electrical synapses [23], and brain regions are connected in functional and anatomical networks [24]. In heterogeneous networks, both nodes and edges can be from different types. For example, in a bibliographic network, nodes in each layer can be authors, venues (journals/conferences) and topics. The nodes can have both intralayer links (e.g. authors write a paper together, which means that there are links between them) and interlayer links (e.g. an author publishes a paper in a specific conference). Multilayer networks may show different statistical and dynamical properties than those with single layers [25–27].

The link prediction problem in multilayer networks is a challenging task. In order to predict a missing or spurious inter- or intralayer link, one needs efficient methods to use the connection information on the target layer as well as other layers. In this work, we consider a two-layer social network and develop an efficient algorithm to predict the forthcoming links in one of the layers. The same individuals make connections in Twitter, a microblogging social network, and Foursquare, a location-based social networking platform. Twitter is a two-sided relation network where users can follow one another to be informed of their latest opinions (tweets). When a user follows another one, it means that there is a directed link between them. Foursquare is a one-sided relation network where users send friendship requests to each other, and when the link initiation is accepted by the requestee, an undirected link is created between the requestor and requestee. In this article, we propose a model based on features which are obtained from these social networks to predict links in Foursquare. The main novelties and contributions of this work are as follows

— we consider multiplex network of a number of users in Twitter and Foursquare, i.e. each network is considered in a separate layer. Twitter layer is extended as a signed network with positive and negative signs as edge labels. A directed link with positive sign from a source node to a destination nodes means that the source node follows the other node, while links with negative sign means that the source node does not follow the destination node. We consider a number of nodal features and examine their influence on the link prediction problem of Foursquare. We use this extended network to calculate two features, namely optimism and reputation, that describe behaviour of nodes in a signed network. Then, we propose a model to predict links in Foursquare network.
— We consider meta-path-based features and examine the influence of the path length on the link prediction problem. A meta-path is a specific type of path in multiplex networks that traverse through the layers and between different types of objects and links. Also, we employ specific type of meta-path, originated from and ending at communities.

## 2. Methods

### 2.1. Background

#### 2.1.1. Link prediction in social networks

Traditionally, social networks have been modelled in a single layer, where all nodes and edges are from the same type. The link prediction problem considers the likelihood of existence of future link between two nodes in a network where there is no link between them in the current state [28]. The link prediction problem has a long history in modern information sciences; however, it was first systematically introduced for mining social networks in [29]. They formulated the problem for a given social network *G* as to predict list of edges not presented in $G[t_{0},\;t_{0}^{\prime }]$, but predicted to exist in $G[t_{1},\;t_{1}^{\prime }]$, where $G[t,\;t^{\prime }]$ denotes the subgraph of *G* at the time-stamp interval of [*t*, *t*′]. $\left[t_{0},\;t_{0}^{\prime }\right]$ is the training interval and $\left[t_{1},\;{t^{\prime }}_{1}\right]$ is testing interval. Hasan *et al*. [30] extended this model and showed that one can obtain better results by employing not only the graph topology, but also contextual information on the nodes and edges. Song *et al*. [31] used matrix factorization to estimate similarity between pair of nodes in a real social network.

Mining heterogeneous networks is more challenging than those with the same type of nodes and links. Link prediction of heterogeneous networks has been studied in some works [32–34]. Hristova *et al*. [35] expressed a number of research questions related to the link prediction problem in heterogeneous networks and proposed a model to solve them. Meta-paths were introduced in [36–38] to analyse heterogeneous networks, extract similarity, predict links and rank their nodes.

#### 2.1.2. Sign prediction problem

The relationships inside social networks can be positive (indicating friendships or trust) or negative (indicating enmity or distrust) [39]. Sign prediction has an important role in social computing applications such as inferring attitude of a specific user towards another user using the information extracted from positive and negative relations that are in the vicinity. Guha *et al*. [40] studied prediction of trust and distrust in the Epinions social network and used propagation algorithms based on exponentiating the adjacency matrix for the prediction process. Leskovec *et al*. [41] used the idea of signed triads and introduced some features based on social and status theories to predict the sign of edges. They further introduced a machine learning framework and showed that it significantly outperforms the social balance and status theories in accurately predicting the signs of the links. Shahriari & Jalili [42] introduced the concepts of reputation and optimism of users and employed them to predict the sign of edges in social networks.

### 2.2. Link prediction in multiplex networks based on meta-paths

In this section, we explain the proposed method for predicting links in multiplex networks. In this work, we model the link prediction problem as a classification task where there are two classes: one class for the existence of future links and another one for non-existence of future links. In order to solve this classification task, one should define a number of features to use in classifiers. Let us define the notation of multiplex networks as follows. A multiplex network is a pair of *M* = (*G*, *C*), where $G=\{G_{\alpha };\alpha \in \{1,2,\ldots ,m\}\}$ (*m* is number of layers) is a family of (directed or undirected, weighted or unweighted) graphs *G_α_* = (*X*_α_, *E_α_*) (called layers of *M*) and

2.1 $$C=\{E_{\alpha \beta }\subseteq X_{\alpha }*X_{\beta };\alpha ,\beta \in \{1,2,\ldots ,m\},\alpha \neq \beta \},$$

is the set of interconnections between nodes of different layers *G_α_* and *G_β_* with *α* ≠ *β*. The elements of *C* are called crossed layers, the elements of each *E_α_* are called intralayer connections of *M* and the elements of each *E_αβ_* (*α* ≠ *β*) are called interlayer connections. *X_α_* is the set of nodes and *E_α_* the set of edges that are present in layer *α*. The dataset in this article consists of two layers which are obtained from two social networks. Thus, $G=\{G_{\alpha };\alpha \in \{T,F\}\},$ where *T* and *F* stand for Twitter and Foursquare, respectively. *G*_T_ is a directed network and *G*_F_ is an undirected one.

In this article, the problem is to predict the links of *G*_F_, having the connectivity information on both *G*_F_ and *G*_T_. To this end, we consider two classes of features. The first class is based on the properties of each node (which is independent from the layers), and the second class of features is based on meta-paths (which depend on the cross-layer paths between the nodes). We explain these features in the following sections.

#### 2.2.1. Node-based features

**Figure 1.**
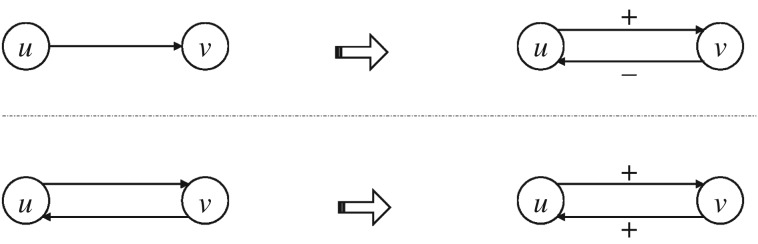
Assigning proper signs to the links in the Twitter network *G*_T_. A directed link is converted to a link in both directions with proper signs (top panel), while a bidirectional link gets positive sign in both directions (bottom panel).

In this section, we explain the nodal features which are used in this article. The node-based features are applied on the Twitter layer (*G*_T_), which is a directed network. In this work, we modify the network structure by assigning proper signs to the links. Any link in *G*_T_ is given a positive or negative sign as follows. Consider two nodes *u* and *v* in *G*_T_. If they follow each other, the edge between them is reciprocal, i.e. there are two directed edges between them (from *u* to *v* and vice versa). In such cases, we put a positive sign on both directions. In the case where one of the nodes follows the other one, but is not being followed, we assign positive sign in the original direction and negative sign in the opposite direction (the negative link does not indeed exist in the network and is artificially made on purpose). Let us suppose *u* follows *v*, but *v* does not follow *u*. We put a positive sign on the edge from *u* to *v* and a negative sign from *v* to *u*. This procedure makes the network signed; however, the edges may have different signs on either direction. Figure 1 shows how signs are given to the edges in *G*_T_.

With the procedure explained above, *G*_T_ becomes a network with both positive and negative signs. There are a number of works in the literature for analysing signed networks and predicting the signs [41–43]. The sign prediction problem in these networks is a special case of the general link prediction problem and is defined as follows. Given the structure (including the edge signs) of a network and *a priori* knowledge on the existence of a link from a source node to a sink node, the problem is to infer the sign of this link. The sign prediction can also be modelled as a classification problem with two classes. Leskovec *et al*. [41] introduced a number of nodal features to use in a machine learning framework for the sign prediction problem. These features are based on node in- and out-degrees and the number of common nodes between the source and sink nodes. A better set of features were proposed by Shahriari & Jalili. [42] where the concepts of reputation and optimism were introduced instead of in- and out-degree. They showed that using reputation and optimism as features significantly improves the sign prediction performance. Reputation refers to the popularity of a node in the social network. As this measure gets higher values for a node, the node becomes more acceptable by others and is likely to be followed. This means that more nodes want to follow this node. The value of reputation for node *u* can be computed taking into account the positive and negative incoming links to the node. Let us denote the number of positive incoming links to *u* by $d_{\mathrm{in}}^{+}(u)$ and the number of negative incoming links to *u* by $d_{\mathrm{in}}^{-}(u)$. The normalized reputation (*RP*) will is computed as [42]

2.2 $$RP=\frac{d_{\mathrm{in}}^{+}(u)-d_{\mathrm{in}}^{-}(u)}{d_{\mathrm{in}}^{+}(u)+d_{\mathrm{in}}^{-}(u)}.$$

Optimism is another feature of a node that can be computed similar to reputation. Nodes with higher values of optimism are likely to point positively to other nodes (i.e. they are likely to follow others). Let us denote the number of positive outgoing links from node *u* to others by $d_{\mathrm{out}}^{+}(u)$ and the number of negative outgoing links by $d_{\mathrm{out}}^{-}(u)$. The normalized optimism (*OP*) of *u* is computed as [42]

2.3 $$OP=\frac{d_{\mathrm{out}}^{+}(u)-d_{\mathrm{out}}^{-}(u)}{d_{\mathrm{out}}^{+}(u)+d_{\mathrm{out}}^{-}(u)}.$$

Here, to predict the link between *u* and *v*, we use common neighbours of these nodes *CN*(*u, v*), which is the number of nodes with links to both *u* and *v*, and their reputation and optimism values *RP*(*u*), *RP*(*v*), *OP*(*u*) and *OP*(*v*), as node-based features from this category.

#### 2.2.2. Meta-path-based features

The above-mentioned features are independent of interlayer information. In this section, we introduce some features to capture the interlayer connectivity information. Our method is based on meta-path strategy [32,33]. A path between two users in a social network has valuable information about their connection such as closeness or friendship. In a multilayer network, two objects can be connected by paths that are crossing different layers of the network or including different types of objects. To account for diverse types of connections among users, a meta-path strategy has been proposed. Different features based on paths between two nodes of homogeneous or heterogeneous networks have been employed to use in the problem of link or sign prediction [32,33,42]. To define meta-path-based features, first, the types of meta-paths between two target objects should be extracted up to a predefined path length based on traversing the target network scheme, using methods such as breadth first search.

**Figure 2.**
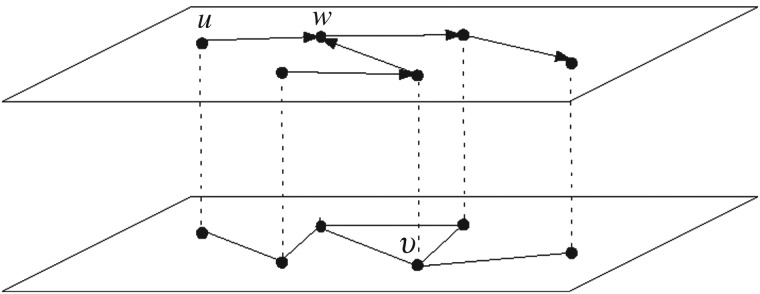
An example of meta-paths between two nodes, starting from node *u*, passing through some objects and ending to node *v*.

Let us formally define a meta-path in our context. Consider two users (nodes) *u* and *v* in the multiplex network. A meta-path with length 2 from *u* in *G*_T_ to *v* in *G*_F_ is shown by $u\overset{\mathrm{follows}}{\to }w\overset{\mathrm{friends}}{\to }v$, which accounts for the relationship between these users; *u* follows *w* in *G*_T_ and *w* is friends with *v* in *G*_F_. Generally, a meta-path between two users consists of many objects in both layers with specific relations. These relations in this domain are {friendship, follows}, where *friendship* is related to *G*_F_ and *follows* shows relations in *G*_T_. To be more precise, consider figure 2 as an illustrating example. It has one meta-path with the above specification; the meta-path from *u* to *v* through *w*. Also, it has two meta-paths with length 3 between node *u* and *v* and one of length 4.

**Figure 3.**
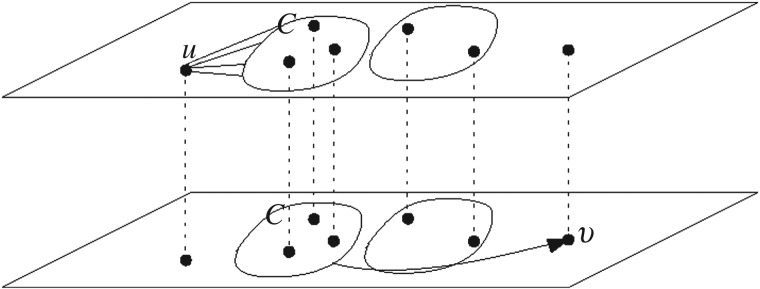
Cluster-based meta-path between two nodes, starting from node *u*, passing through cluster *C*, (any node that belongs to cluster *C*) and ending to node *v*.

As mentioned above, meta-paths have valuable information about connection of nodes in heterogeneous networks. To define meaningful features to use in the classification task of the link prediction problem, we should determine the length and type of relations which are used in a meta-path between two users. Finding a meta-path can be done by traversing network scheme using methods such as breadth first search. After finding the meta-paths, meaningful features should be extracted from them. Sun & Han [33] introduced various features based on meta-paths such as path counts. Path counts try to extend the concept of ‘strong and weak ties’ to multilayer networks, accounting for the strength of connectedness between two users. In this article, we use meta-paths with different length (2–4) as classification features. Real social networks datasets are often highly sparse, which makes machine learning methods (i.e. classifiers) have poor performance. In other words, for highly sparse connections in the layers, one might not find common neighbouring nodes for many pairs (for any *u* and *v* from different layers, there should be a *w* connecting them (figure 2), which is not the case for many pairs). Javari *et al*. [34] proposed a novel method to overcome this problem by employing meta-paths based on the clusters in the layer. To this end, instead of computing a meta-path between two users based on the other objects in the layers, they employed clusters to propose a new definition of meta-path. In signed networks, a cluster is referred to a group of nodes for which the intracluster links are positive as much as possible, whereas negative links are placed within cluster as much as possible [44,45]. This is obtained by minimizing intercluster positive links and intracluster negative connections. In the approach introduced in [34], denoted by cluster-based meta-path, a meta-path with length 2 between nodes *u* and *v* in the network, is shown by $u\overset{\mathrm{follows}}{\to }C\overset{\mathrm{friends}}{\to }v$, which states the relation between two users and one cluster *C* between them. This means that user *u* follows a node in cluster *C* in *G*_T_ and a node in cluster *C* is friends with *v* in *G*_F_. Figure 3 illustrates the cluster-based meta-path methodology.

Therefore, we employ the count of cluster-based meta-paths (CB-MP) with length 2, 3 and 4 as features in our formulation of the problem. In order to extract the community structure in the layer, we use InfoMap [46,47], which is a well-known community detection algorithm. For every pair of nodes *u* and *v*, we calculate all CB-MPs of different lengths that pass through each cluster in *G*_T_ and have one of the following conditions: (*i* is 1, 2 or 3)

(1) A path from node *u* to a cluster *C* has at most a length *i* and located in *G*_T_. Also, there exists a path from cluster *C* with length 1 that is placed in *G*_F_.
(2) A path from node *u* to a cluster *C* has length 1 and is located in *G*_F_. Also, there exists a path from cluster *C* with at most length *i* and is placed in *G*_T_.

We consider three different lengths for the CB-MPs along with the above mutable conditions, thus obtaining a set of six features for any pair of nodes.

### 2.3. Data description

In this article, we consider a two-layer social network, with social connections between the same users in Twitter and Foursquare networks. In Twitter network, users can share their short messages (tweets), which should be at most 140 characters. The users can follow each other to have interaction with followee's tweets. This network is directed, where the links are created from followers to followees. It frequently happens that a user follows another user, but not being followed by the same user. In Foursquare, users can check-in every place. Users' check-ins in Foursquare share their opinion about venues or their mobility with their friends. This network is undirected, meaning that when a user accepts a friendship request made by another user, there is an undirected friendship link between them.

To construct this multiplex network, we crawled 45 000 users in both Twitter and Foursquare. Then, we applied modified breadth first search and depth first search to find the largest connected component and all paths between objects in the network. Next, we could find a set of 1508 users that were present in both networks. We then filtered the networks and considered the connectivity information between these 1508 users in these networks. Table 1 represents demographic information of these networks. Foursquare network is denser than Twitter network. We study the interlayer dependencies in these networks. Studying the interlayer relations gives insights into the structural dependencies between the layers and how to design a model to maximally use the available information for the inference tasks. Here, Kendall's *τ*-rank correlation coefficient is used to analyse interlayer degree correlation, normalized Hamming distance is employed to measure the interlayer correlation of node activities, means follower/following edges in Twitter and friendship in Foursquare, and the number of common edges is used to study the interlayer correlation of edge presence. Despite these metrics being coarse-grained, they can represent an overall summary of the correlations of important structural patterns in the multiplex network. Kendall's *τ*-rank correlation coefficient, as a value between −1 and 1, measures the degree correlation between the layers. Here, higher Kendall's *τ* correlation coefficient means that nodes who are friends in Foursquare layer, follow each other and are followed by each other in Twitter layer. As it can be seen in table 2, this coefficient is 0.49, which indicates moderate correlations between the layers in this sense. Also, we calculate Hamming distance measure between Twitter and Foursquare layers. A higher value of this measure reveals that there is no dependency between activities of the nodes. We find high Hamming distance values indicating low dependency between the layers. There are many common edges in the layers; about 43% of the edges of Twitter network are also present in Foursquare network.

**Table 1.** Statistics of the multilayer network studied in this work.

network	no. nodes	no. edges	average degree	clustering coefficient
Twitter	1508	15 161	in = 10.05, out = 10	0.21
Foursquare	1508	18 471	24.4	0.34

**Table 2.** Correlations of the layers (*G*_T_ and *G*_F_) in terms of various metrics.

	Kendall's τ correlation coefficient		Hamming distance		
pair of layers	in-degree	out-degree	in-activity	out-activity	no. common edges
*G*_T_− *G*_F_	0.49	0.38	0.91	0.94	6546

### 2.4. Classification methods

As the node-based and meta-path-based features are extracted, classical classifiers are used to perform the link prediction task, which is indeed a two-class classification problem. Here we choose support vector machines (SVMs) [48], naive Bayes [49] and K-nearest neighbour (KNN) [50] as classifiers. SVM constructs a set of hyperplanes in a high dimensional space, and different classes are optimally separated by optimizing the distance to the functional margins. Because many real-world problems are not linearly separable, the original finite dimensional space is mapped to a much higher dimensional space, using a kernel function. Here we use SVM with a Gaussian kernel.

Naive Bayes is a simple and effective classification method. It constructs a family of a number of algorithms that assume the value of a specific feature is independent of other features. Maximum-likelihood estimation is used to train the parameters of the models. KNN is another classification method used in this work. It is a non-parametric classification method in which the input consists of the *K* closest training samples in the feature space, and the function is approximated locally. The classification is performed by computing a weighted average of the functions such that the nearer neighbours contribute more to the average than the more distal ones. Here we optimize *K* in the range [1,50] to train KNN classifier.

### 2.5. Classical link prediction methods

We compare the performance of machine-learning-based methods with a number of baseline link prediction methods proposed for single-layer networks [3,4]. These baseline methods include common neighbour coefficient (CN), Jaccard coefficient (JC), Adamic Adar coefficient (AA) and preferential attachment coefficient (PA). These methods are similarity-based techniques that define a similarity index between any two nodes, and the higher the similarity the higher the probability of creating links between them. CN is the number of common neighbours for two nodes. JC normalizes CN by dividing the total number of neighbours. AA is obtained by summing inverse of logarithm of CN's degrees. PA computes similarity between two nodes by multiplying their degrees.

### 2.6. Assessment metrics

In order to assess the performance of the predictors, we use different evaluation metrics including accuracy, root mean square error (RMSE), mean average error (MAE) and the receiver operating characteristic (ROC). Accuracy is true results (true positives and true negatives) divided by the total number of cases. RMSE is the standard deviation of the differences between the actual values and the predicted ones. MAE is the average absolute difference between the actual and predicted values. ROC is a graphical plot to illustrate the performance of binary classification systems. It plots the false positives against the true positives.

## 3. Results and discussions

In this section, we apply the proposed method on the above two-layer network to predict undirected links in Foursquare layer. In other words, the connection structure of Twitter and Foursquare networks are used to predict the links in Foursquare. All simulations were performed using Matlab and its associated toolboxes. We compare the performance of these classifiers on four sets of features (NB, MP2, MPs and NB/MPs). In node-based (NB) feature set, the common neighbours for the end nodes along with their optimism and reputation values are considered. In meta-path 2 (MP2) feature set, we use the counts of meta-path with length 2, whereas in MPs meta-paths of lengths 2, 3 and 4 are considered. In NB/MPs, all features are considered together.

Because we have only two classes in the dataset—class zero for non-existing links and class one for the existing links—this prediction problem is a binary problem. The original version of this multiplex network is unbalanced, i.e. there are many more links from class zero when compared with class one. This unbalanced distribution of the labels might result in poor performance for the prediction model. To avoid this, we should make this dataset balanced by choosing equal size of data for the two classes. Thus, we use all links in class one and randomly select an equal number of links from class zero. This process is repeated 20 time, and the average results are reported.

**Figure 4.**
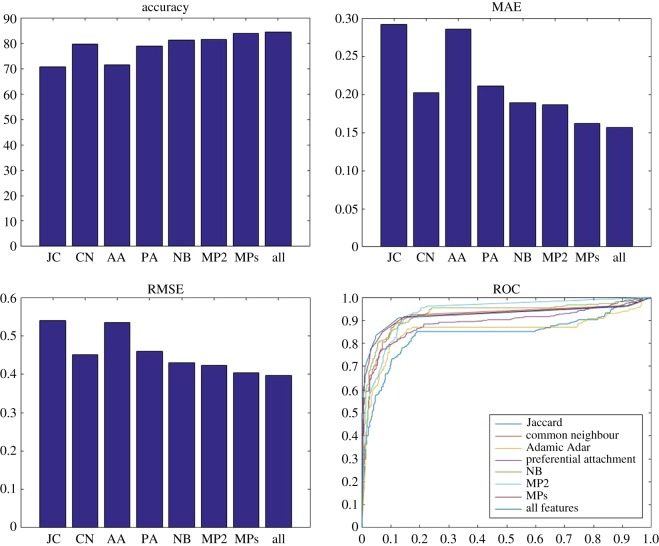
Accuracy, MAE, RMSE and ROC for the balanced version of the dataset. Naive Bayes is used as a classifier and eight different sets (Jaccard coefficient (JC), common neighbour coefficient (CN), Adamic Adar coefficient (AA), preferential attachment coefficient (PA), node-based feature (NB), meta-path with length 2 (MP2), meta-path with lengths 2, 3 and 4 (MPs) and NB/MPs) as features. JC and AA result in the worst performance, whereas MPs and NB/MPs have the best performance.

**Figure 5.**
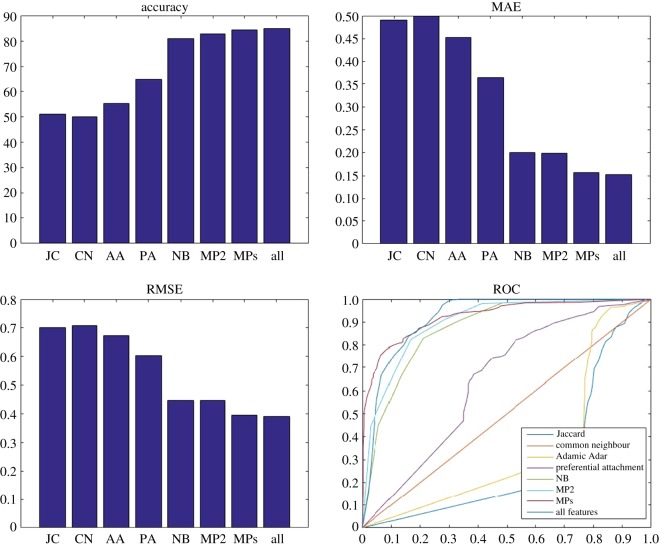
Accuracy, MAE, RMSE and ROC for the balanced version of the dataset. K-nearest neighbour is used as a classifier and eight different sets (JC, CN, AA, PA, NB, MP2, MPs and NB/MPs) as features. NB, MP2, MPs and NB/MPs have significantly better performance than other methods, whereas MPs and NB/MPs are the top performers.

**Figure 6.**
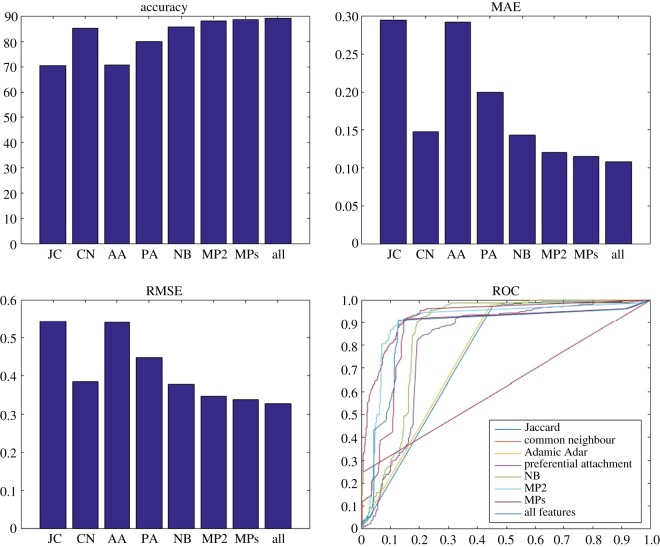
Accuracy, MAE, RMSE and ROC for the balanced version of the dataset. SVM is used as a classifier and eight different sets (JC, CN, AA, PA,NB, MP2, MPs and NB/MPs) as features. JC and AA result in the worst performance, whereas MPs and NB/MPs have the best performance.

Figures 4–6 show the results of the link prediction using different classifiers. Figure 4 shows the performance of naive Bayes using the four feature sets extracted from the balanced datasets. The NB/MPs feature set is the top performer with accuracy of 84%, whereas baseline methods have the poorest performance with accuracy of around 75%. Considering the meta-path-based features (MP2 and MPs) significantly improves the prediction performance (with about 5% higher accuracy) when compared with NB features. However, increasing the length of the meta-paths does not significantly improve the performance, although the computational complexity dramatically increases. This indicates that to predict the links in one of the layers, considering the information of other layers improves the prediction performance.

Figure 5 illustrates the results when K-nearest neighbour is used as a classifier. Our proposed link prediction method significantly improves the results (about 35% decreased MAE, 30% decreased RMSE and 34% increased accuracy) when compared with the baseline methods. The best performance is obtained when all features are included in the classification task (NB/MPs). SVM is also used as a classifier and the results are shown in figure 6. Again, NB/MPs is the top performer, while baseline methods have the poorest performance. Our proposed method with this classifier has accuracy of 90%, whereas baseline methods have accuracy of 80% in the best case. SVM shows a better performance (around 5%) against KNN and naive Bayes. Generally, Our NB/MPs model has the best performance in comparison with other models and various classifiers.

## 4. Conclusion

The link prediction problem in social networks has many potential applications. Examples include predicting forthcoming links from users to items in recommendation systems, friendship suggestions and discovering spurious connections. Recently, a new paradigm of network modelling has been proposed within the community of network science. Many real networks including online social networks evolve their connections in different layers (e.g. different social networking platforms); such networks are denoted by multiplex networks. Network with multiple layers might show completely different behaviour than those with only a single layer of entities. In this article, we studied the link prediction problem in a real two-layer social network. The same individuals have connections in two social networks (Twitter and Foursquare), with one network being a directed network and another being undirected. Here we used the structural information of the layers (both Twitter and Foursquare networks) to predict the links in Foursquare network. To this end, two sets of features were considered: those based on nodal properties, which are independent of the interlayer information, and those based on meta-paths, which consider cross-layer information. We also used three classical classifiers to solve the prediction task. Our experiments showed that although the layers do not have high interlayer correlations, including cross-layer information could significantly improve the prediction performance. Although here we focused on a specific definition of the link prediction problem by considering the likelihood of the existence of future links given that such links are not currently present, similar formalism can be used to study missing links, which requires to have the structure of the networks in different times.

## Supplementary Material

The ESM zip contains three files. The file fedges.txt are the edges that define the network, the file tedges.txt are the edges between the different layers of the network, while data in the file twitter_foursquare_mapper.dat provides the basic info of each node of the network, as stated in the first row.

## Data accessibility

The datasets supporting this article have been uploaded as part of the supplementary material. twitter_foursquare_mapper.dat: mapping between Twitter usernames and Foursquare IDs. fedges.txt: connectivity of users in Foursquare. tedges.txt: connectivity of users in Twitter.
